# (25*R*)-6α-Hy­droxy-5α-spiro­stan-3β-yl tosyl­ate

**DOI:** 10.1107/S1600536812046600

**Published:** 2012-11-24

**Authors:** María A. Fernández-Herrera, Jesús Sandoval-Ramírez, Sylvain Bernès, Maricela Rodríguez-Acosta, María-Guadalupe Hernández Linares

**Affiliations:** a Benemérita Universidad Autónoma de Puebla, Facultad de Ciencias Químicas, Ciudad Universitaria, Puebla, Pue. 72570, Mexico; b Universidad Autónoma de Nuevo León, UANL, Facultad de Ciencias Químicas, Av. Universidad S/N, Ciudad Universitaria, San Nicolás de los Garza, Nuevo León CP 66451, Mexico; c Herbario y Jardín Botánico, Benemérita Universidad Autónoma de Puebla, Ciudad Universitaria, Puebla, Pue. 72570, Mexico; d Escuelas de Ingeniería en Petróleos e Ingeniería Química, Universidad del Istmo, Ciudad Universitaria s/n, 70760 Sto. Domingo Tehuantepec, Oax., Mexico

## Abstract

The title steroid, C_34_H_50_O_6_S, is an inter­mediate on the synthetic route between diosgenin and brassinosteroids, which possess the *A* ring modified with the 2α,3α-diol functionality. The polycyclic spiro­stan system has the expected conformation, with six-membered rings adopting chair forms and the five-membered rings envelope forms (flap atoms are the methine C atom in the *C*/*D*-ring junction and the spiro C atom connecting rings *E* and *F*). The 3β-tosyl­ate group is oriented in such a way that S=O bonds are engaged in inter­molecular hydrogen bonds with O—H and C—H donors. Chains of mol­ecules are formed along [100] *via* O—H⋯O hydrogen bonds, and secondary weak C—H⋯O inter­actions connect two neighbouring chains in the [001] direction.

## Related literature

For background to brassinosteroids, see: Asami *et al.* (2005 ▶); Kang & Guo (2011 ▶); Zullo & Adam (2002 ▶). For the hydro­boration-oxidation synthetic step used for the preparation of the title compound, see: Smith & Pelter (1991 ▶); Brown (1962 ▶). For the structure of another steroid functionalized at C-3 with a tosyl­ate group, see: Cox *et al.* (1996 ▶).
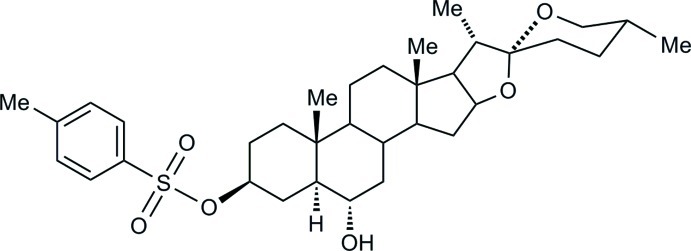


## Experimental

### 

#### Crystal data

C_34_H_50_O_6_S
*M* *_r_* = 586.80
Orthorhombic, 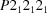

*a* = 6.7653 (8) Å
*b* = 12.2856 (11) Å
*c* = 37.943 (4) Å
*V* = 3153.6 (6) Å^3^
*Z* = 4
Mo *K*α radiation
μ = 0.15 mm^−1^
*T* = 296 K
0.6 × 0.5 × 0.4 mm

#### Data collection

Bruker P4 diffractometer
Absorption correction: ψ scan (*XSCANS*; Siemens, 1996 ▶) *T*_min_ = 0.905, *T*_max_ = 0.943
9218 measured reflections
6167 independent reflections
5274 reflections with *I* > 2σ(*I*)
*R*_int_ = 0.023
3 standard reflections every 97 reflections intensity decay: 1.5%

#### Refinement

*R*[*F* ^2^ > 2σ(*F* ^2^)] = 0.036
*wR*(*F* ^2^) = 0.090
*S* = 1.03
6167 reflections
380 parameters
H atoms treated by a mixture of independent and constrained refinement
Δρ_max_ = 0.16 e Å^−3^
Δρ_min_ = −0.16 e Å^−3^
Absolute structure: Flack (1983 ▶), 2504 Friedel pairs
Flack parameter: −0.03 (7)

### 

Data collection: *XSCANS* (Siemens, 1996 ▶); cell refinement: *XSCANS*; data reduction: *XSCANS*; program(s) used to solve structure: *SHELXS97* (Sheldrick, 2008 ▶); program(s) used to refine structure: *SHELXL97* (Sheldrick, 2008 ▶); molecular graphics: *SHELXTL* (Sheldrick, 2008 ▶); software used to prepare material for publication: *SHELXL97*.

## Supplementary Material

Crystal structure: contains datablock(s) I, global. DOI: 10.1107/S1600536812046600/bq2379sup1.cif

Structure factors: contains datablock(s) I. DOI: 10.1107/S1600536812046600/bq2379Isup2.hkl

Additional supplementary materials:  crystallographic information; 3D view; checkCIF report

## Figures and Tables

**Table 1:** Hydrogen-bond geometry (Å, °)

*D*—H⋯*A*	*D*—H	H⋯*A*	*D*⋯*A*	*D*—H⋯*A*
O30—H30⋯O33^i^	0.78 (3)	2.35 (3)	3.098 (2)	159 (3)
C40—H40*A*⋯O34^ii^	0.93	2.65	3.352 (3)	133

Symmetry codes: (i) 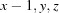; (ii) 
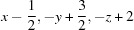
.

**Fig. 1.**
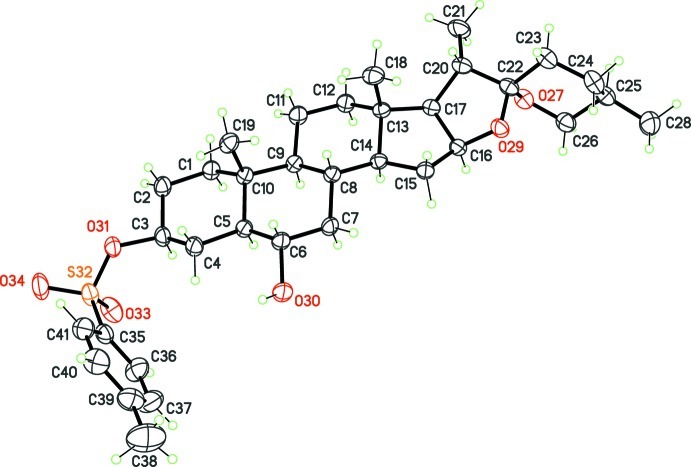
ORTEP view of the title molecule, with displacement ellipsoids at the 30% probability level.

**Fig. 2.**
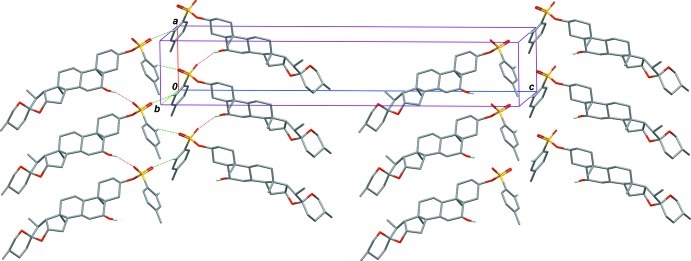
Part of the crystal structure of the title compound, showing pairs of chains. On the left stack, intermolecular contacts are depicted with dashed bonds. Red: O—H···O hydrogen bonds; green: interchain C—H···O weak interactions.

## supplementary crystallographic information

### Comment

Brassinosteroids (BS) are endogenous plant hormones essential for the regulation of multiple physiological processes required for normal plant growth and development (Asami *et al.*, 2005). Since their discovery, more than 30 years ago, the synthetic chemistry has been extensively developed for obtaining BS and analogs (Zullo & Adam, 2002; Kang & Guo, 2011). The most active BS and analogs possess a 2α,3α-diol in ring *A* and a ketone at C-6 (or a lactone) in ring *B*. The introduction of the required 6-keto-2α,3α-diol functionality has been satisfactorily achieved from the 6-hydroxy-3β-tosylate framework. The title compound belongs to this line of synthetic approaches to BS analogs. It was synthesized from diosgenin, through a tosylation followed by hydroboration-oxidation (Smith & Pelter, 1991; Brown, 1962). Commonly, the crude product after the hydroboration-oxidation procedure is immediately oxidized in order to obtain the 6-keto derivative; we decided instead, to isolate and properly characterize the 6-hydroxy intermediate.

The compound crystallizes with one molecule in the asymmetric unit (Fig. 1) and the conformation of the *A*-*F* ring system is as expected for a spirostan nucleus. All 6-membered rings have a chair conformation, while 5-membered rings *D* and *E* are envelopes on C14 and C22, respectively. The tosylate group in equatorial position at C3 is oriented in such a way that a potential intramolecular stabilizing O—H···π contact could be formed between the hydroxyl group at C6 and the benzene ring of the tosylate. However, this interaction should have an energy approaching zero, because of the too long H···π separation, *ca*. 4.4 Å. On the other hand, the tosylate orientation in the title compound is similar to that observed in cholesteryl tosylate (Cox *et al.*, 1996), which has C-6 engaged in a double bond. This suggests that the tosylate orientation results from packing restraints or intermolecular interactions rather than intramolecular contacts.

Regarding the crystal structure, the single feature of interest is the intermolecular hydrogen bond formed between the hydroxyl group and one S═O group in the tosylate. These contacts link molecules in chains oriented in the [100] direction in the crystal. A weak hydrogen bond involving the other S═O group is observed between chains, C40—H40*A*···O34, characterized by a small C—H···O angle of 133° (Fig. 2).

### Experimental

Diosgenin (750 mg, 1.8 mmol) was tosylated by means of *p*-TsCl/py/DCM, following the standard procedure, affording diosgenin tosylate quantitatively; the crude was properly washed, dried and immediately submitted to the next reaction. Diosgenin tosylate (1 g, 1.76 mmol) was dissolved in THF (30 ml) and NaBH_4_ (0.4 g, 10.8 mmol) was added. The system was sealed under Ar atmosphere and then, BF_3_^.^Et_2_O (0.7 ml, 5.6 mmol) was carefully added. The reaction mixture was kept for 2 h at room temperature, concentrated under reduced pressure, and re-dissolved in a solution of KOH/MeOH (2%, 50 ml), followed by 5 ml of 35% H_2_O_2_. The reaction mixture was stirred for 1 h; then the addition of water produced a precipitate, which was filtered off, washed with cold water, and dried under high vacuum. The resulting white powder was purified by column chromatography with hexanes/EtOAc 7:3 to afford the title compound as a white powder. It was recrystallized from hexanes/EtOAc 8:2 to obtain 0.72 g (70%) of colourless crystals. *M*.p. 149–150 °C, [α]_D_ -39° (*c* 1.0, CHCl_3_). Spectroscopic characterization may be found in the archived CIF.

### Refinement

Hydroxyl H atom H30 was found in a difference map and refined with free coordinates and isotropic *U* parameter. Other H atoms were placed in idealized positions and refined with a riding model and fixed isotropic *U* parameters. C—H bond lengths were fixed to 0.96 (methyl), 0.97 (methylene), or 0.98 Å (methine). Displacement parameters were calculated as *U*_iso_(H) = *xU*_eq_(parent C) where *x* = 1.5 (methyl) or 1.2 (methylene, methine). Anomalous dispersion of the tosylate S atom allowed to refine a Flack parameter (Flack, 1983), which is in agreement with the expected absolute configuration for the molecule.

### Crystal data

**Table:**

C_34_H_50_O_6_S	*D*_x_ = 1.236 Mg m^−^^3^
*M**_r_* = 586.80	Melting point: 422 K
Orthorhombic, *P*2_1_2_1_2_1_	Mo *K*α radiation, λ = 0.71073 Å
Hall symbol: P 2ac 2ab	Cell parameters from 72 reflections
*a* = 6.7653 (8) Å	θ = 4.6–12.5°
*b* = 12.2856 (11) Å	µ = 0.15 mm^−^^1^
*c* = 37.943 (4) Å	*T* = 296 K
*V* = 3153.6 (6) Å^3^	Prism, colourless
*Z* = 4	0.6 × 0.5 × 0.4 mm
*F*(000) = 1272	

### Data collection

**Table:**

Bruker P4 diffractometer	5274 reflections with *I* > 2σ(*I*)
Radiation source: fine-focus sealed tube, FN4	*R*_int_ = 0.023
Graphite monochromator	θ_max_ = 26.3°, θ_min_ = 2.0°
ω scans	*h* = −8→6
Absorption correction: ψ scan (*XSCANS*; Siemens, 1996)	*k* = −15→15
*T*_min_ = 0.905, *T*_max_ = 0.943	*l* = −46→47
9218 measured reflections	3 standard reflections every 97 reflections
6167 independent reflections	intensity decay: 1.5%

### Refinement

**Table:**

Refinement on *F*^2^	Hydrogen site location: inferred from neighbouring sites
Least-squares matrix: full	H atoms treated by a mixture of independent and constrained refinement
*R*[*F*^2^ > 2σ(*F*^2^)] = 0.036	*w* = 1/[σ^2^(*F*_o_^2^) + (0.0359*P*)^2^ + 0.5535*P*] where *P* = (*F*_o_^2^ + 2*F*_c_^2^)/3
*wR*(*F*^2^) = 0.090	(Δ/σ)_max_ = 0.002
*S* = 1.03	Δρ_max_ = 0.16 e Å^−^^3^
6167 reflections	Δρ_min_ = −0.16 e Å^−^^3^
380 parameters	Extinction correction: *SHELXL97* (Sheldrick, 2008), Fc^*^=kFc[1+0.001xFc^2^λ^3^/sin(2θ)]^-1/4^
0 restraints	Extinction coefficient: 0.0058 (5)
0 constraints	Absolute structure: Flack (1983), 2504 Friedel pairs
Primary atom site location: structure-invariant direct methods	Flack parameter: −0.03 (7)
Secondary atom site location: difference Fourier map	

### Fractional atomic coordinates and isotropic or equivalent isotropic displacement parameters (Å^2^)

**Table:**

	*x*	*y*	*z*	*U*_iso_*/*U*_eq_	
C1	0.7277 (3)	0.76613 (17)	0.81367 (5)	0.0487 (5)	
H1A	0.7549	0.7024	0.7994	0.058*	
H1B	0.8056	0.8259	0.8044	0.058*	
C2	0.7923 (3)	0.74351 (17)	0.85158 (5)	0.0535 (5)	
H2A	0.7246	0.6793	0.8603	0.064*	
H2B	0.9333	0.7293	0.8521	0.064*	
C3	0.7455 (3)	0.83933 (17)	0.87512 (5)	0.0501 (5)	
H3A	0.8291	0.9013	0.8686	0.060*	
C4	0.5302 (3)	0.87241 (18)	0.87337 (5)	0.0510 (5)	
H4A	0.5095	0.9374	0.8874	0.061*	
H4B	0.4485	0.8148	0.8830	0.061*	
C5	0.4702 (3)	0.89471 (15)	0.83500 (4)	0.0424 (4)	
H5A	0.5563	0.9532	0.8265	0.051*	
C6	0.2580 (3)	0.93700 (16)	0.83213 (5)	0.0478 (4)	
H6A	0.1664	0.8805	0.8402	0.057*	
C7	0.2094 (3)	0.96671 (16)	0.79427 (5)	0.0484 (5)	
H7A	0.2901	1.0282	0.7872	0.058*	
H7B	0.0720	0.9889	0.7929	0.058*	
C8	0.2445 (3)	0.87236 (14)	0.76861 (4)	0.0392 (4)	
H8A	0.1540	0.8130	0.7748	0.047*	
C9	0.4581 (3)	0.82992 (14)	0.77203 (4)	0.0397 (4)	
H9A	0.5443	0.8920	0.7668	0.048*	
C10	0.5071 (3)	0.79483 (14)	0.81045 (4)	0.0398 (4)	
C11	0.5084 (3)	0.74214 (17)	0.74437 (5)	0.0520 (5)	
H11A	0.6494	0.7280	0.7451	0.062*	
H11B	0.4410	0.6752	0.7507	0.062*	
C12	0.4505 (3)	0.77331 (17)	0.70650 (5)	0.0493 (5)	
H12A	0.5339	0.8326	0.6985	0.059*	
H12B	0.4723	0.7116	0.6910	0.059*	
C13	0.2344 (3)	0.80791 (14)	0.70437 (4)	0.0392 (4)	
C14	0.2068 (3)	0.90418 (14)	0.73031 (4)	0.0394 (4)	
H14A	0.3094	0.9573	0.7243	0.047*	
C15	0.0107 (3)	0.95550 (16)	0.71905 (5)	0.0487 (4)	
H15A	−0.0063	1.0275	0.7291	0.058*	
H15B	−0.1011	0.9102	0.7255	0.058*	
C16	0.0397 (3)	0.96013 (15)	0.67900 (4)	0.0444 (4)	
H16A	0.1065	1.0281	0.6726	0.053*	
C17	0.1717 (3)	0.86180 (14)	0.66890 (4)	0.0409 (4)	
H17A	0.2900	0.8885	0.6567	0.049*	
C18	0.1006 (3)	0.71110 (16)	0.71335 (5)	0.0555 (5)	
H18A	0.1350	0.6833	0.7362	0.083*	
H18B	0.1178	0.6550	0.6960	0.083*	
H18C	−0.0348	0.7344	0.7135	0.083*	
C19	0.3831 (3)	0.69510 (15)	0.82133 (5)	0.0517 (5)	
H19A	0.4338	0.6313	0.8099	0.078*	
H19B	0.2480	0.7062	0.8145	0.078*	
H19C	0.3902	0.6858	0.8464	0.078*	
C20	0.0414 (3)	0.79983 (16)	0.64246 (5)	0.0483 (5)	
H20A	−0.0400	0.7484	0.6558	0.058*	
C21	0.1537 (4)	0.7352 (2)	0.61469 (6)	0.0721 (7)	
H21A	0.0614	0.7007	0.5991	0.108*	
H21B	0.2333	0.6808	0.6260	0.108*	
H21C	0.2373	0.7834	0.6015	0.108*	
C22	−0.0941 (3)	0.88812 (16)	0.62844 (4)	0.0471 (4)	
C23	−0.2867 (3)	0.85149 (19)	0.61189 (5)	0.0588 (5)	
H23A	−0.3665	0.8151	0.6296	0.071*	
H23B	−0.2587	0.7995	0.5933	0.071*	
C24	−0.4025 (4)	0.9461 (2)	0.59666 (6)	0.0648 (6)	
H24A	−0.5165	0.9185	0.5840	0.078*	
H24B	−0.4499	0.9918	0.6157	0.078*	
C25	−0.2772 (4)	1.01361 (18)	0.57190 (5)	0.0593 (5)	
H25A	−0.2429	0.9686	0.5515	0.071*	
C26	−0.0883 (4)	1.04441 (18)	0.59073 (5)	0.0599 (5)	
H26A	−0.0032	1.0834	0.5745	0.072*	
H26B	−0.1198	1.0932	0.6100	0.072*	
O27	0.0167 (2)	0.95231 (12)	0.60433 (3)	0.0537 (3)	
C28	−0.3833 (4)	1.1154 (2)	0.55875 (6)	0.0824 (7)	
H28A	−0.2911	1.1613	0.5465	0.124*	
H28B	−0.4377	1.1543	0.5784	0.124*	
H28C	−0.4878	1.0948	0.5430	0.124*	
O29	−0.1410 (2)	0.95014 (11)	0.65939 (3)	0.0510 (3)	
O30	0.2301 (3)	1.03360 (13)	0.85290 (4)	0.0693 (5)	
H30	0.206 (5)	1.017 (3)	0.8723 (8)	0.107 (12)*	
O31	0.7980 (2)	0.80181 (12)	0.91112 (3)	0.0565 (4)	
S32	0.89495 (8)	0.88025 (5)	0.938758 (13)	0.05819 (15)	
O33	1.0188 (2)	0.95749 (16)	0.92101 (4)	0.0753 (5)	
O34	0.9767 (3)	0.80926 (16)	0.96460 (4)	0.0825 (5)	
C35	0.6942 (3)	0.94918 (16)	0.95735 (5)	0.0506 (5)	
C36	0.6646 (4)	1.05871 (19)	0.95077 (7)	0.0735 (7)	
H36A	0.7537	1.0974	0.9369	0.088*	
C37	0.5013 (5)	1.1100 (2)	0.96490 (8)	0.0859 (8)	
H37A	0.4812	1.1834	0.9601	0.103*	
C38	0.3682 (4)	1.0562 (2)	0.98576 (7)	0.0763 (7)	
C39	0.4014 (4)	0.9468 (2)	0.99195 (6)	0.0749 (7)	
H39A	0.3125	0.9084	1.0059	0.090*	
C40	0.5620 (3)	0.89296 (19)	0.97808 (5)	0.0614 (6)	
H40A	0.5811	0.8193	0.9827	0.074*	
C41	0.1927 (6)	1.1145 (3)	1.00114 (11)	0.1337 (14)	
H41A	0.1886	1.1877	0.9923	0.201*	
H41B	0.2040	1.1160	1.0264	0.201*	
H41C	0.0737	1.0771	0.9946	0.201*	

### Atomic displacement parameters (Å^2^)

**Table:**

	*U*^11^	*U*^22^	*U*^33^	*U*^12^	*U*^13^	*U*^23^
C1	0.0473 (11)	0.0556 (11)	0.0431 (10)	0.0123 (9)	0.0071 (9)	0.0001 (9)
C2	0.0517 (12)	0.0623 (12)	0.0465 (10)	0.0189 (10)	0.0007 (9)	0.0011 (9)
C3	0.0565 (12)	0.0576 (11)	0.0362 (9)	0.0088 (10)	−0.0011 (9)	0.0058 (8)
C4	0.0581 (12)	0.0573 (11)	0.0378 (9)	0.0136 (10)	0.0044 (8)	0.0005 (9)
C5	0.0475 (10)	0.0443 (9)	0.0355 (8)	0.0061 (9)	0.0048 (8)	0.0013 (7)
C6	0.0553 (11)	0.0487 (10)	0.0394 (9)	0.0171 (9)	0.0063 (9)	−0.0048 (8)
C7	0.0552 (12)	0.0494 (10)	0.0407 (9)	0.0202 (10)	0.0017 (8)	−0.0024 (8)
C8	0.0428 (10)	0.0393 (9)	0.0356 (8)	0.0050 (8)	0.0057 (7)	−0.0007 (7)
C9	0.0406 (10)	0.0420 (9)	0.0367 (8)	0.0025 (8)	0.0077 (7)	0.0027 (7)
C10	0.0411 (10)	0.0416 (9)	0.0369 (8)	0.0078 (8)	0.0084 (8)	0.0022 (7)
C11	0.0537 (11)	0.0586 (11)	0.0436 (10)	0.0190 (10)	0.0077 (9)	−0.0053 (9)
C12	0.0533 (12)	0.0549 (11)	0.0397 (9)	0.0111 (10)	0.0076 (8)	−0.0040 (9)
C13	0.0441 (10)	0.0369 (8)	0.0366 (8)	−0.0013 (8)	0.0069 (8)	−0.0022 (7)
C14	0.0446 (10)	0.0357 (9)	0.0380 (8)	−0.0003 (8)	0.0050 (8)	−0.0008 (7)
C15	0.0578 (12)	0.0491 (10)	0.0393 (9)	0.0138 (10)	−0.0017 (9)	−0.0074 (8)
C16	0.0529 (11)	0.0416 (9)	0.0387 (9)	−0.0013 (9)	−0.0036 (8)	−0.0019 (8)
C17	0.0439 (10)	0.0418 (9)	0.0371 (8)	−0.0051 (8)	0.0046 (7)	−0.0016 (7)
C18	0.0685 (13)	0.0443 (10)	0.0536 (11)	−0.0121 (11)	0.0078 (11)	0.0016 (9)
C19	0.0589 (12)	0.0464 (10)	0.0498 (10)	0.0020 (10)	0.0090 (10)	0.0082 (9)
C20	0.0554 (12)	0.0471 (10)	0.0424 (9)	−0.0055 (9)	0.0033 (8)	−0.0089 (8)
C21	0.0797 (17)	0.0773 (16)	0.0594 (13)	0.0107 (14)	−0.0034 (12)	−0.0307 (12)
C22	0.0501 (10)	0.0540 (10)	0.0373 (8)	−0.0082 (10)	0.0034 (8)	−0.0058 (8)
C23	0.0579 (13)	0.0719 (14)	0.0468 (11)	−0.0172 (12)	−0.0006 (10)	−0.0029 (10)
C24	0.0556 (13)	0.0872 (16)	0.0516 (11)	−0.0077 (14)	−0.0082 (10)	−0.0018 (11)
C25	0.0699 (14)	0.0672 (13)	0.0409 (10)	−0.0005 (12)	−0.0053 (10)	−0.0072 (9)
C26	0.0707 (14)	0.0584 (12)	0.0506 (11)	−0.0120 (12)	−0.0046 (11)	0.0041 (10)
O27	0.0522 (8)	0.0616 (8)	0.0472 (7)	−0.0106 (7)	0.0010 (6)	0.0036 (7)
C28	0.0959 (19)	0.0858 (17)	0.0654 (14)	0.0142 (17)	−0.0129 (14)	0.0027 (14)
O29	0.0533 (8)	0.0609 (8)	0.0387 (6)	0.0071 (7)	−0.0027 (6)	−0.0085 (6)
O30	0.0981 (13)	0.0661 (9)	0.0435 (8)	0.0378 (10)	0.0019 (9)	−0.0110 (7)
O31	0.0708 (10)	0.0598 (8)	0.0390 (6)	0.0138 (8)	−0.0043 (6)	0.0051 (6)
S32	0.0559 (3)	0.0777 (4)	0.0410 (2)	0.0079 (3)	−0.0039 (2)	0.0055 (2)
O33	0.0600 (9)	0.1110 (13)	0.0551 (9)	−0.0139 (10)	0.0071 (8)	0.0017 (9)
O34	0.0882 (12)	0.1071 (13)	0.0521 (8)	0.0312 (11)	−0.0188 (9)	0.0102 (9)
C35	0.0605 (12)	0.0548 (11)	0.0365 (9)	−0.0038 (10)	0.0006 (9)	0.0038 (9)
C36	0.0881 (19)	0.0585 (13)	0.0740 (15)	−0.0034 (13)	0.0191 (13)	0.0177 (12)
C37	0.108 (2)	0.0530 (13)	0.0968 (18)	0.0096 (16)	0.0092 (18)	0.0002 (14)
C38	0.0751 (17)	0.0809 (17)	0.0730 (15)	0.0027 (15)	0.0090 (13)	−0.0160 (14)
C39	0.0727 (16)	0.0827 (17)	0.0692 (14)	−0.0126 (15)	0.0197 (13)	0.0031 (13)
C40	0.0720 (15)	0.0563 (12)	0.0559 (11)	−0.0085 (12)	0.0084 (11)	0.0065 (10)
C41	0.117 (3)	0.134 (3)	0.150 (3)	0.031 (3)	0.041 (3)	−0.040 (3)

### Geometric parameters (Å, º)

**Table:**

C1—C2	1.528 (3)	C18—H18B	0.9600
C1—C10	1.539 (3)	C18—H18C	0.9600
C1—H1A	0.9700	C19—H19A	0.9600
C1—H1B	0.9700	C19—H19B	0.9600
C2—C3	1.511 (3)	C19—H19C	0.9600
C2—H2A	0.9700	C20—C22	1.516 (3)
C2—H2B	0.9700	C20—C21	1.523 (3)
C3—O31	1.485 (2)	C20—H20A	0.9800
C3—C4	1.514 (3)	C21—H21A	0.9600
C3—H3A	0.9800	C21—H21B	0.9600
C4—C5	1.536 (2)	C21—H21C	0.9600
C4—H4A	0.9700	C22—O27	1.422 (2)
C4—H4B	0.9700	C22—O29	1.435 (2)
C5—C6	1.530 (3)	C22—C23	1.515 (3)
C5—C10	1.561 (2)	C23—C24	1.516 (3)
C5—H5A	0.9800	C23—H23A	0.9700
C6—O30	1.437 (2)	C23—H23B	0.9700
C6—C7	1.518 (2)	C24—C25	1.513 (3)
C6—H6A	0.9800	C24—H24A	0.9700
C7—C8	1.532 (2)	C24—H24B	0.9700
C7—H7A	0.9700	C25—C26	1.512 (3)
C7—H7B	0.9700	C25—C28	1.526 (3)
C8—C14	1.526 (2)	C25—H25A	0.9800
C8—C9	1.542 (2)	C26—O27	1.432 (3)
C8—H8A	0.9800	C26—H26A	0.9700
C9—C11	1.543 (2)	C26—H26B	0.9700
C9—C10	1.556 (2)	C28—H28A	0.9600
C9—H9A	0.9800	C28—H28B	0.9600
C10—C19	1.541 (3)	C28—H28C	0.9600
C11—C12	1.538 (3)	O30—H30	0.78 (3)
C11—H11A	0.9700	O31—S32	1.5680 (15)
C11—H11B	0.9700	S32—O34	1.4241 (16)
C12—C13	1.525 (3)	S32—O33	1.4340 (18)
C12—H12A	0.9700	S32—C35	1.749 (2)
C12—H12B	0.9700	C35—C40	1.377 (3)
C13—C18	1.533 (3)	C35—C36	1.383 (3)
C13—C14	1.550 (2)	C36—C37	1.380 (4)
C13—C17	1.559 (2)	C36—H36A	0.9300
C14—C15	1.529 (3)	C37—C38	1.369 (4)
C14—H14A	0.9800	C37—H37A	0.9300
C15—C16	1.533 (2)	C38—C39	1.383 (4)
C15—H15A	0.9700	C38—C41	1.504 (4)
C15—H15B	0.9700	C39—C40	1.377 (3)
C16—O29	1.436 (2)	C39—H39A	0.9300
C16—C17	1.550 (3)	C40—H40A	0.9300
C16—H16A	0.9800	C41—H41A	0.9600
C17—C20	1.537 (3)	C41—H41B	0.9600
C17—H17A	0.9800	C41—H41C	0.9600
C18—H18A	0.9600		
			
C2—C1—C10	113.18 (16)	C16—C17—C13	105.90 (13)
C2—C1—H1A	108.9	C20—C17—H17A	109.1
C10—C1—H1A	108.9	C16—C17—H17A	109.1
C2—C1—H1B	108.9	C13—C17—H17A	109.1
C10—C1—H1B	108.9	C13—C18—H18A	109.5
H1A—C1—H1B	107.8	C13—C18—H18B	109.5
C3—C2—C1	110.78 (16)	H18A—C18—H18B	109.5
C3—C2—H2A	109.5	C13—C18—H18C	109.5
C1—C2—H2A	109.5	H18A—C18—H18C	109.5
C3—C2—H2B	109.5	H18B—C18—H18C	109.5
C1—C2—H2B	109.5	C10—C19—H19A	109.5
H2A—C2—H2B	108.1	C10—C19—H19B	109.5
O31—C3—C2	104.59 (15)	H19A—C19—H19B	109.5
O31—C3—C4	110.72 (16)	C10—C19—H19C	109.5
C2—C3—C4	112.62 (18)	H19A—C19—H19C	109.5
O31—C3—H3A	109.6	H19B—C19—H19C	109.5
C2—C3—H3A	109.6	C22—C20—C21	115.60 (17)
C4—C3—H3A	109.6	C22—C20—C17	102.79 (15)
C3—C4—C5	110.11 (15)	C21—C20—C17	115.06 (18)
C3—C4—H4A	109.6	C22—C20—H20A	107.6
C5—C4—H4A	109.6	C21—C20—H20A	107.6
C3—C4—H4B	109.6	C17—C20—H20A	107.6
C5—C4—H4B	109.6	C20—C21—H21A	109.5
H4A—C4—H4B	108.2	C20—C21—H21B	109.5
C6—C5—C4	112.09 (15)	H21A—C21—H21B	109.5
C6—C5—C10	112.00 (15)	C20—C21—H21C	109.5
C4—C5—C10	112.53 (15)	H21A—C21—H21C	109.5
C6—C5—H5A	106.6	H21B—C21—H21C	109.5
C4—C5—H5A	106.6	O27—C22—O29	110.39 (15)
C10—C5—H5A	106.6	O27—C22—C23	110.60 (16)
O30—C6—C7	106.97 (15)	O29—C22—C23	107.87 (16)
O30—C6—C5	111.38 (17)	O27—C22—C20	107.67 (16)
C7—C6—C5	110.63 (15)	O29—C22—C20	103.08 (14)
O30—C6—H6A	109.3	C23—C22—C20	116.93 (18)
C7—C6—H6A	109.3	C22—C23—C24	111.99 (18)
C5—C6—H6A	109.3	C22—C23—H23A	109.2
C6—C7—C8	112.68 (15)	C24—C23—H23A	109.2
C6—C7—H7A	109.1	C22—C23—H23B	109.2
C8—C7—H7A	109.1	C24—C23—H23B	109.2
C6—C7—H7B	109.1	H23A—C23—H23B	107.9
C8—C7—H7B	109.1	C25—C24—C23	111.6 (2)
H7A—C7—H7B	107.8	C25—C24—H24A	109.3
C14—C8—C7	112.66 (14)	C23—C24—H24A	109.3
C14—C8—C9	108.86 (14)	C25—C24—H24B	109.3
C7—C8—C9	110.35 (15)	C23—C24—H24B	109.3
C14—C8—H8A	108.3	H24A—C24—H24B	108.0
C7—C8—H8A	108.3	C26—C25—C24	108.51 (17)
C9—C8—H8A	108.3	C26—C25—C28	110.3 (2)
C8—C9—C11	112.69 (16)	C24—C25—C28	112.9 (2)
C8—C9—C10	111.83 (14)	C26—C25—H25A	108.3
C11—C9—C10	113.38 (15)	C24—C25—H25A	108.3
C8—C9—H9A	106.1	C28—C25—H25A	108.3
C11—C9—H9A	106.1	O27—C26—C25	113.06 (17)
C10—C9—H9A	106.1	O27—C26—H26A	109.0
C1—C10—C19	108.94 (16)	C25—C26—H26A	109.0
C1—C10—C9	110.15 (14)	O27—C26—H26B	109.0
C19—C10—C9	110.82 (16)	C25—C26—H26B	109.0
C1—C10—C5	106.74 (16)	H26A—C26—H26B	107.8
C19—C10—C5	112.22 (14)	C22—O27—C26	114.12 (16)
C9—C10—C5	107.90 (14)	C25—C28—H28A	109.5
C12—C11—C9	113.93 (15)	C25—C28—H28B	109.5
C12—C11—H11A	108.8	H28A—C28—H28B	109.5
C9—C11—H11A	108.8	C25—C28—H28C	109.5
C12—C11—H11B	108.8	H28A—C28—H28C	109.5
C9—C11—H11B	108.8	H28B—C28—H28C	109.5
H11A—C11—H11B	107.7	C22—O29—C16	106.34 (13)
C13—C12—C11	111.28 (15)	C6—O30—H30	109 (2)
C13—C12—H12A	109.4	C3—O31—S32	121.63 (13)
C11—C12—H12A	109.4	O34—S32—O33	120.11 (12)
C13—C12—H12B	109.4	O34—S32—O31	104.27 (10)
C11—C12—H12B	109.4	O33—S32—O31	109.71 (9)
H12A—C12—H12B	108.0	O34—S32—C35	108.69 (10)
C12—C13—C18	109.76 (16)	O33—S32—C35	108.83 (11)
C12—C13—C14	107.14 (15)	O31—S32—C35	104.03 (9)
C18—C13—C14	112.31 (14)	C40—C35—C36	119.8 (2)
C12—C13—C17	115.15 (15)	C40—C35—S32	119.47 (16)
C18—C13—C17	111.15 (16)	C36—C35—S32	120.73 (17)
C14—C13—C17	101.04 (13)	C37—C36—C35	119.4 (2)
C8—C14—C15	121.10 (15)	C37—C36—H36A	120.3
C8—C14—C13	112.90 (14)	C35—C36—H36A	120.3
C15—C14—C13	103.99 (14)	C38—C37—C36	122.1 (2)
C8—C14—H14A	105.9	C38—C37—H37A	119.0
C15—C14—H14A	105.9	C36—C37—H37A	119.0
C13—C14—H14A	105.9	C37—C38—C39	117.4 (2)
C14—C15—C16	100.44 (15)	C37—C38—C41	120.9 (3)
C14—C15—H15A	111.7	C39—C38—C41	121.7 (3)
C16—C15—H15A	111.7	C40—C39—C38	122.0 (2)
C14—C15—H15B	111.7	C40—C39—H39A	119.0
C16—C15—H15B	111.7	C38—C39—H39A	119.0
H15A—C15—H15B	109.5	C39—C40—C35	119.4 (2)
O29—C16—C15	113.67 (16)	C39—C40—H40A	120.3
O29—C16—C17	107.18 (14)	C35—C40—H40A	120.3
C15—C16—C17	106.85 (14)	C38—C41—H41A	109.5
O29—C16—H16A	109.7	C38—C41—H41B	109.5
C15—C16—H16A	109.7	H41A—C41—H41B	109.5
C17—C16—H16A	109.7	C38—C41—H41C	109.5
C20—C17—C16	102.53 (15)	H41A—C41—H41C	109.5
C20—C17—C13	120.61 (15)	H41B—C41—H41C	109.5
			
C10—C1—C2—C3	−56.5 (2)	O29—C16—C17—C13	−127.69 (15)
C1—C2—C3—O31	174.77 (17)	C15—C16—C17—C13	−5.49 (19)
C1—C2—C3—C4	54.5 (2)	C12—C13—C17—C20	107.2 (2)
O31—C3—C4—C5	−171.61 (16)	C18—C13—C17—C20	−18.4 (2)
C2—C3—C4—C5	−54.9 (2)	C14—C13—C17—C20	−137.79 (17)
C3—C4—C5—C6	−175.57 (17)	C12—C13—C17—C16	−137.32 (16)
C3—C4—C5—C10	57.1 (2)	C18—C13—C17—C16	97.08 (17)
C4—C5—C6—O30	56.6 (2)	C14—C13—C17—C16	−22.27 (17)
C10—C5—C6—O30	−175.81 (15)	C16—C17—C20—C22	24.43 (18)
C4—C5—C6—C7	175.44 (17)	C13—C17—C20—C22	141.67 (16)
C10—C5—C6—C7	−57.0 (2)	C16—C17—C20—C21	150.96 (18)
O30—C6—C7—C8	176.66 (17)	C13—C17—C20—C21	−91.8 (2)
C5—C6—C7—C8	55.2 (2)	C21—C20—C22—O27	−50.2 (2)
C6—C7—C8—C14	−176.75 (17)	C17—C20—C22—O27	76.01 (17)
C6—C7—C8—C9	−54.8 (2)	C21—C20—C22—O29	−166.88 (18)
C14—C8—C9—C11	−50.40 (19)	C17—C20—C22—O29	−40.70 (18)
C7—C8—C9—C11	−174.53 (15)	C21—C20—C22—C23	75.0 (2)
C14—C8—C9—C10	−179.51 (15)	C17—C20—C22—C23	−158.82 (16)
C7—C8—C9—C10	56.36 (19)	O27—C22—C23—C24	−52.4 (2)
C2—C1—C10—C19	−64.9 (2)	O29—C22—C23—C24	68.4 (2)
C2—C1—C10—C9	173.36 (16)	C20—C22—C23—C24	−176.07 (17)
C2—C1—C10—C5	56.5 (2)	C22—C23—C24—C25	52.5 (2)
C8—C9—C10—C1	−173.24 (15)	C23—C24—C25—C26	−52.1 (2)
C11—C9—C10—C1	58.0 (2)	C23—C24—C25—C28	−174.71 (18)
C8—C9—C10—C19	66.14 (19)	C24—C25—C26—O27	54.8 (2)
C11—C9—C10—C19	−62.6 (2)	C28—C25—C26—O27	178.89 (18)
C8—C9—C10—C5	−57.08 (19)	O29—C22—O27—C26	−63.9 (2)
C11—C9—C10—C5	174.17 (16)	C23—C22—O27—C26	55.4 (2)
C6—C5—C10—C1	175.76 (15)	C20—C22—O27—C26	−175.73 (15)
C4—C5—C10—C1	−56.9 (2)	C25—C26—O27—C22	−58.3 (2)
C6—C5—C10—C19	−65.0 (2)	O27—C22—O29—C16	−73.14 (18)
C4—C5—C10—C19	62.4 (2)	C23—C22—O29—C16	165.92 (16)
C6—C5—C10—C9	57.39 (19)	C20—C22—O29—C16	41.62 (18)
C4—C5—C10—C9	−175.26 (16)	C15—C16—O29—C22	−143.50 (16)
C8—C9—C11—C12	48.6 (2)	C17—C16—O29—C22	−25.66 (18)
C10—C9—C11—C12	176.91 (17)	C2—C3—O31—S32	142.58 (15)
C9—C11—C12—C13	−52.6 (2)	C4—C3—O31—S32	−95.9 (2)
C11—C12—C13—C18	−64.8 (2)	C3—O31—S32—O34	−162.55 (15)
C11—C12—C13—C14	57.4 (2)	C3—O31—S32—O33	−32.69 (18)
C11—C12—C13—C17	168.88 (15)	C3—O31—S32—C35	83.60 (16)
C7—C8—C14—C15	−53.7 (2)	O34—S32—C35—C40	−42.7 (2)
C9—C8—C14—C15	−176.44 (16)	O33—S32—C35—C40	−175.16 (17)
C7—C8—C14—C13	−177.83 (15)	O31—S32—C35—C40	67.93 (18)
C9—C8—C14—C13	59.4 (2)	O34—S32—C35—C36	138.8 (2)
C12—C13—C14—C8	−63.21 (19)	O33—S32—C35—C36	6.4 (2)
C18—C13—C14—C8	57.4 (2)	O31—S32—C35—C36	−110.5 (2)
C17—C13—C14—C8	175.91 (15)	C40—C35—C36—C37	−0.5 (4)
C12—C13—C14—C15	163.71 (15)	S32—C35—C36—C37	177.9 (2)
C18—C13—C14—C15	−75.69 (18)	C35—C36—C37—C38	0.9 (4)
C17—C13—C14—C15	42.83 (17)	C36—C37—C38—C39	−0.8 (4)
C8—C14—C15—C16	−174.43 (16)	C36—C37—C38—C41	179.3 (3)
C13—C14—C15—C16	−46.22 (17)	C37—C38—C39—C40	0.4 (4)
C14—C15—C16—O29	149.34 (15)	C41—C38—C39—C40	−179.7 (3)
C14—C15—C16—C17	31.31 (18)	C38—C39—C40—C35	−0.1 (4)
O29—C16—C17—C20	−0.41 (18)	C36—C35—C40—C39	0.1 (3)
C15—C16—C17—C20	121.79 (16)	S32—C35—C40—C39	−178.35 (18)

### Hydrogen-bond geometry (Å, º)

**Table:**

*D*—H···*A*	*D*—H	H···*A*	*D*···*A*	*D*—H···*A*
O30—H30···O33^i^	0.78 (3)	2.35 (3)	3.098 (2)	159 (3)
C40—H40*A*···O34^ii^	0.93	2.65	3.352 (3)	133

Symmetry codes: (i) *x*−1, *y*, *z*; (ii) *x*−1/2, −*y*+3/2, −*z*+2.

## References

[bb1] Asami, T., Nakano, T. & Fujioka, S. (2005). *Vitam. Horm.* **72**, 479–504. 10.1016/S0083-6729(05)72014-8 16492480

[bb2] Brown, H. C. (1962). *Hydroboration*, pp. 12–13. New York: W. A. Benjamin Inc.

[bb3] Cox, P. J., Buchanan, H. J. & Wardell, J. L. (1996). *Acta Cryst.* C**52**, 2111–2113.

[bb4] Flack, H. D. (1983). *Acta Cryst.* A**39**, 876–881.

[bb5] Kang, Y. Y. & Guo, S. R. (2011). *Brassinosteroids: A Class of Plant Hormone*, edited by S. Hayat & A. Ahmad, pp. 269–288. Dordrecht: Springer.

[bb6] Sheldrick, G. M. (2008). *Acta Cryst.* A**64**, 112–122. 10.1107/S0108767307043930 18156677

[bb7] Siemens (1996). *XSCANS* Siemens Analytical X-ray Instruments Inc., Madison, Wisconsin, USA.

[bb8] Smith, K. & Pelter, A. (1991). *Comprehensive Organic Synthesis*, Vol. 8, edited by B. M. Trost & I. Fleming, pp. 703–731. Oxford: Pergamon Press.

[bb9] Zullo, M. A. T. & Adam, G. (2002). *Braz. J. Plant Physiol* **14**, 143–181.

